# Quality Control of Gamma Irradiated Dwarf Mallow (*Malva neglecta* Wallr.) Based on Color, Organic Acids, Total Phenolics and Antioxidant Parameters

**DOI:** 10.3390/molecules21040467

**Published:** 2016-04-08

**Authors:** José Pinela, Lillian Barros, Amilcar L. Antonio, Ana Maria Carvalho, M. Beatriz P. P. Oliveira, Isabel C. F. R. Ferreira

**Affiliations:** 1 Mountain Research Centre (CIMO), ESA, Polytechnic Institute of Bragança, Campus de Santa Apolónia, 1172, 5300-253 Bragança, Portugal; jpinela@ipb.pt (J.P.); lillian@ipb.pt (L.B.); amilcar@ipb.pt (A.L.A.); anacarv@ipb.pt (A.M.C.); 2 REQUIMTE/LAQV, Faculty of Pharmacy, University of Porto, Rua Jorge Viterbo Ferreira, no. 228, 4050-313 Porto, Portugal; beatoliv@ff.up.pt; 3 Centro de Ciências e Tecnologias Nucleares (C2TN), Instituto Superior Técnico, Universidade de Lisboa, E.N. 10, 2695-066 Bobadela, Portugal

**Keywords:** *Malva neglecta*, ionizing radiation, color parameters, organic acids, antioxidant activity, decoction, quality control

## Abstract

This study addresses the effects of gamma irradiation (1, 5 and 8 kGy) on color, organic acids, total phenolics, total flavonoids, and antioxidant activity of dwarf mallow (*Malva neglecta* Wallr.). Organic acids were analyzed by ultra fast liquid chromatography (UFLC) coupled to a photodiode array (PDA) detector. Total phenolics and flavonoids were measured by the Folin-Ciocalteu and aluminium chloride colorimetric methods, respectively. The antioxidant activity was evaluated based on the DPPH^•^ scavenging activity, reducing power, β-carotene bleaching inhibition and thiobarbituric acid reactive substances (TBARS) formation inhibition. Analyses were performed in the non-irradiated and irradiated plant material, as well as in decoctions obtained from the same samples. The total amounts of organic acids and phenolics recorded in decocted extracts were always higher than those found in the plant material or hydromethanolic extracts, respectively. The DPPH^•^ scavenging activity and reducing power were also higher in decocted extracts. The assayed irradiation doses affected differently the organic acids profile. The levels of total phenolics and flavonoids were lower in the hydromethanolic extracts prepared from samples irradiated at 1 kGy (dose that induced color changes) and in decocted extracts prepared from those irradiated at 8 kGy. The last samples also showed a lower antioxidant activity. In turn, irradiation at 5 kGy favored the amounts of total phenolics and flavonoids. Overall, this study contributes to the understanding of the effects of irradiation in indicators of dwarf mallow quality, and highlighted the decoctions for its antioxidant properties.

## 1. Introduction

Organic acids are primary metabolites widely spread throughout the plant kingdom. Chemically, they are low weight molecules and are considered to be any organic carboxylic acid with a general structure R-COOH. Although they are weak acids, these water-soluble compounds may confer acidic properties to foods containing them and influence its organoleptic properties (flavor, color and aroma) and consequent acceptability by the consumers [1]. From a practical point of view, the organic acids profile (levels and relative ratios) is important for food quality control. It allows determining the percentage of plant added to final products, thus detecting adulterations or possible microbial deteriorations occurring during storage, providing a practical advantage for its use as an authenticity index in plant-based foods and beverages [1,2,3]. The relative amounts and the presence of each of these compounds are also useful as a means to evaluate the effect of food processing [2,4]. Thus, qualitative and quantitative analyses of these ingredients are of great importance.

During the production process (from harvesting and drying to packaging and storage), raw plants are prone to various contaminations and infestations, which can lead to spoilage, quality deterioration and consequent economic losses [5]. Besides constituting health hazards to consumers, contaminated products can also adversely affect the efficacy and stability of their bioactive ingredients and lead to spoilage of final products [6]. The presence of organic acids and phenolic compounds is advantageous, as they contribute to the natural preservation process (through its antimicrobial and antioxidant activities), but is not enough. The search for new preservation treatments with a minimum impact on physical, chemical and functional parameters highlighted the gamma irradiation as a safe, effective and sustainable option to sanitize plant products [7,8].

Nowadays there is a growing scientific interest in irradiation-induced modifications of antioxidant properties and compounds responsible for such effects. The antioxidant activity is strongly linked the presence of phenolic compounds [9,10,11,12], secondary metabolites frequently found attached to sugars (glycosides), which increases their water solubility. These compounds have the ability to donate a hydrogen atom from the aromatic hydroxyl group to a free radical and/or the capacity to support an unpaired electron in their aromatic structures. Therefore, the methods used to evaluate the antioxidant activity can be classified according to the mechanism of action, *i.e.*, single-electron transfer or hydrogen atom transfer [13]. However, phenolic compounds, organic acids and other bioactive constituents may be affected by irradiation treatment if applied inappropriately. It is known that ionizing radiation interacts with water molecules generating free radicals, in a reaction commonly known as radiolysis [14]. These free radicals can then interact with different biomolecules, leading to breakdown of chemical bonds and changes in their structure and, consequently, in the bioactivity and extractability from the plant material. The referred compounds may also be affected by the direct impact of the gamma-rays [14,15]. As a consequence, the color of the processed samples may change. However, this quality attribute is directly related to consumers’ appreciation of a product, as they tend to associate its color with its taste, hygienic safety, shelf-life and personal satisfaction [16,17]. A very stringent plant selection based on color parameters also occurs in the food, pharmaceutical and cosmeceutical industries [18]. Therefore, the color evaluation is important in quality control parameter of irradiated products.

Dwarf mallow (*Malva neglecta* Wallr.) is an annual herbaceous plant of the family Malvaceae traditionally eaten raw as a leafy vegetable or prepared in herbal beverages (mainly decoctions) due to claims of disinfectant and anti-inflammatory effects [19]. It is also used to treat multiple medical conditions such as asthma, colds, digestive and urinary problems, and abdominal pains [20]. Scientific works have reported antioxidant [11,20], antibacterial [21] and anti-ulcerogenic [22] properties for this plant. Nevertheless, as far as we know, the organic acids profile of this plant remains unknown, as well as the effects induced by the gamma irradiation treatment on its physical or chemical properties. Thus, the main purpose of this study was to investigate the dose-response effects of gamma irradiation on color, organic acids, total phenolics and flavonoids, and antioxidant properties (quality control indicators) of *M. neglecta* samples. The influence of the preparation method (decoction) was also investigated.

## 2. Results

### 2.1. Effects on Color Parameters

**Figure 1:**
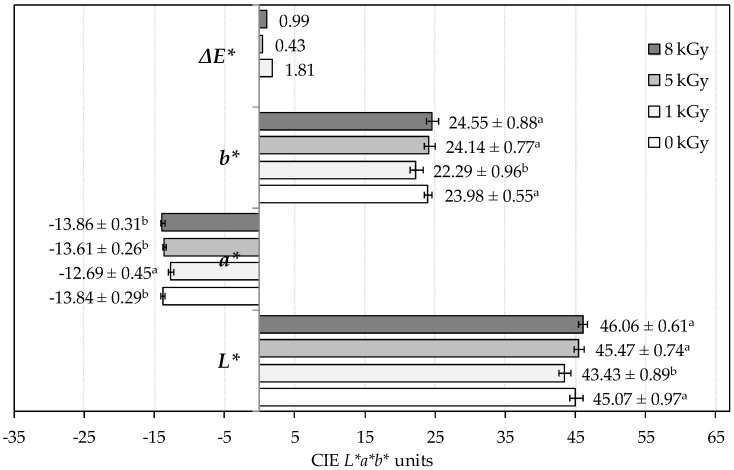
Impact of the gamma irradiation treatment on color parameters of *M. neglecta* powdered samples. ΔE*: total color difference; b*: blueness (–) ↔ yellowness (+); a*: greenness (–) ↔ redness (+); and L*: darkness (0) ↔ lightness (100). For each color parameter, different letters (a,b) indicate statistically significant differences (*p* < 0.05).

The CIE L*a*b* color values for non-irradiated and irradiated samples of *M. neglecta* are presented in Figure 1. The 5 and 8 kGy doses had no influence on color parameters. However, significant differences were found for samples irradiated with the 1 kGy dose. These samples revealed lower lightness (L* = 43.43 ± 0.89) and yellowness (b* = 22.29 ± 0.96) and higher redness (a* = −12.69 ± 0.45), which induced the more pronounced total color difference (ΔE* = 1.81), compared with the non-irradiated control samples (L* = 45.07 ± 0.97, b* = 23.98 ± 0.55 and a* = −13.84 ± 0.29). Furthermore, while the ΔE* up to 0.5 and from 0.5 to 1.5 indicate trace color differences and slight differences, respectively, the ΔE* corresponding to the 1 kGy dose indicates a noticeable difference detectable by the human eye [23].

Similar results were previously reported by Kirkin *et al.* [24]. Those authors found that the 7 kGy dose had a greater effect on color parameters of *Rosmarinus officinalis* L. than higher doses of 12 and 17 kGy. In these samples, the L* value was decreased and the a* value was increased, in accordance to our results. The discoloration of *Piper nigrum* L. by decreasing the a* and b* values and increasing the L* value was also reported, as well as the suitability of the applied doses to maintain the color parameters of *Thymus vulgaris* L. and *Cuminum cyminum* L. A tendency for lightness decrease was also reported by Pereira *et al.* [25] in *Ginkgo biloba* L. samples irradiated at 10 kGy. Nevertheless, browning is unwanted in dried products [26]. On the other hand, Pinela *et al.* [9] reported that gamma irradiation treatment up to 10 kGy had no effect on the color parameters of shade- and freeze-dried samples of *Tuberaria lignosa* (Sweet) Samp. Therefore, we can conclude that the irradiation-induced modifications on color parameters not only depend on the applied dose, but also on the plant material under investigation.

### 2.2. Effects on Organic Acids

**Figure 2:**
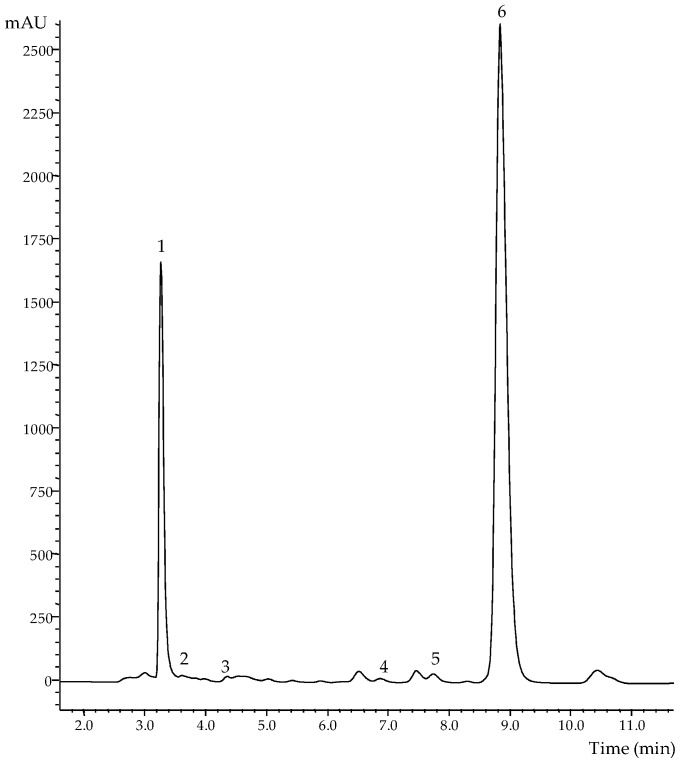
Chromatographic profile of organic acids in the decoction prepared from *M. neglecta* samples irradiated at 5 kGy, obtained using UFLC-PDA. Peak identification: 1: oxalic acid, 2: quinic acid, 3: malic acid, 4: citric acid, 5: succinic acid, and 6: fumaric acid.

Table 1 shows the organic acids content found in *M. neglecta* dry material submitted to gamma irradiation, as well as in the decocted extracts (the form traditionally used in folk medicine) prepared from that material. Oxalic, quinic, malic, citric, succinic and fumaric acids were quantified, being oxalic acid the most abundant organic acid (Figure 2). The total amounts recorded in the decocted extracts were always higher than those found in the plant dry material, as well as the individual levels of each organic acid (except for citric acid) (Table 1). This difference may be related to a better extraction efficiency achieved at elevated temperature, but that can lead to citric acid degradation [27]. These results are in agreement with those previously reported by Guimarães *et al.* [10] for *Matricaria recutita* L. dry material and decocted extracts.

In the plant material, the relative amounts of oxalic, citric and fumaric acids were the most affected by the irradiation treatment (Table 1); however, this variation was more marked in the decocted extracts, mainly in the oxalic and succinic acids, probably due to the combined effects induced by gamma irradiation and preparation method. Therefore, the levels of organic acids and relative ratios in the decocted extracts were somewhat different from those found in the dry material, e.g., citric acid was the second most abundant organic acid in the plant material and presented a reduction of ~12% in the decocted extracts, while the relative ratios of succinic acid increased ~19% in those preparations. Nevertheless, although oxalic, malic, citric and fumaric acids have been already described in flowers and flowering shoots of *Malva sylvestris* L. [28], quinic and succinic acids were not detected or present in trace amounts in this species, so a qualitative analysis of the organic acids profile can be used to detect possible adulterations in the samples.

In the dry material, the 1 kGy dose did not induce any adverse effect on the organic acids profile and increased the total levels. Lower amounts of total organic acids were however detected in samples irradiated at 5 kGy, due to a decrease of oxalic and fumaric acids. The highest levels of citric and succinic acids and a reduction of fumaric acid detected in both samples (dry material and decocted extract) were also associated with the 5 kGy dose. Finally, the samples irradiated at 8 kGy gave the highest levels of total organic acids. It can be concluded that, in general, the effects induced by the different doses were positive, as the decrease in total organic acids was mainly associated with the oxalic acid content. Despite the importance of this acid in the pharmaceutical industry [29], it has been associated with some health problems, e.g., oxalic acid can combine with calcium in the kidneys to form kidney stones in susceptible people [30].

The identified organic acids have been used as food additives or pharmaceutical and cosmetic excipients [31,32]. Citric acid is a widely used food additive in many kinds of beverages due to its mild and refreshing sourness [1,33], succinic acid is also used in the food industry as well as in pharmaceuticals and antibiotics [31], fumaric acid is used due to its effectiveness against psoriasis and inflammation and due to its neuro- and chemoprotective effects [34], and malic and citric acids are reported to have bactericidal effects [35]. Additionally, the consumption of these compounds in moderate amounts can promote appetite, help digestion and be beneficial to human health [4]. Nevertheless, excessive doses of certain organic acids should be avoided [32].

### 2.3. Effects on Total Phenolics, Total Flavonoids and Antioxidant Activity

The effects of gamma irradiation on the total phenolic and flavonoid contents and antioxidant activity of the *M. neglecta* hydromethanolic and decocted extracts can be accessed from the analysis of Table 2. The antioxidant activity results are expressed in EC_50_ values; thus, the lower the EC_50_ value, the higher the antioxidant activity. In general, the decocted extracts revealed higher amounts of total phenolics and flavonoids than the hydromethanolic extracts, as well as an increased DPPH^•^ scavenging activity and reducing power. The samples irradiated at 1 kGy and extracted with the hydromethanolic mixture revealed lower levels of both total phenolic and flavonoids and decreased DPPH^•^ scavenging activity, reducing power, and TBARS formation inhibition capacity. This result is in agreement with the higher ΔE* found in these samples. Contrariwise, the hydromethanolic extracts prepared from the samples irradiated at 5 kGy revealed higher levels of total phenolics and flavonoids and an increased DPPH^•^ scavenging activity and lipid peroxidation inhibition capacity (accessed by the β-carotene bleaching inhibition and TBARS formation inhibition in brain cell homogenates) in comparison with those prepared from the non-irradiated control samples. In fact, the lipid peroxidation inhibition capacity of these extracts was favored by the irradiation treatment (except for the TBARS formation inhibition of the extracts prepared from the samples irradiated at 1 kGy). This result can be explained by the affinity of these two *in vitro* assays for lipophilic antioxidants and by the fact that the irradiation treatment affects the different antioxidant molecules differently [14,15].

Regarding the decocted extracts, the 8 kGy dose decreased the antioxidant properties and levels of total phenolics and flavonoids. However, the other assayed doses reinforced the reducing power, the TBARS formation inhibition capacity, and the levels of total phenolics and flavonoids. Similar trends have been observed in other studies. A higher DPPH^•^ scavenging activity and reducing power of decocted and infused extracts of *T. vulgaris*, compared to the hydroalcoholic ones, was found by Martins *et al.* [12]. Decoction was also the preferable preparation method for obtaining increased levels of phenolic acids and flavonoids. The suitability of decoctions and infusions for extracting phenolic compounds from commercial samples of *Achillea millefolium* L. was demonstrated by Dias *et al.* [36], who prepared aqueous extracts that also had interesting antioxidant properties. Furthermore, despite the plant extracts rich in phenolic compounds are commonly referred to as having increased antioxidant properties [9,10,11], the presence of organic acids, as phenolics, may also contribute to these effects [37].

The total phenolic and flavonoid contents of different parts of *M. neglecta* were already reported by Dalar *et al.* [11] for samples from the Eastern Anatolia Region of Turkey. Lower total phenolic contents of 17.4 ± 0.3 mg GAE/g extract and 6.6 ± 0.3 mg GAE/g extract were reported in leaf and whole plant extracts, respectively, obtained using acidified methanol (80% methanol and 1% HCl (*v*/*v*) in water) as extraction solvent. Regarding total flavonoids, 7.21 ± 0.28 mg RE (rutin equivalents)/g extract and 2.95 ± 0.16 mg RE/g extract were found in the leaf and whole plant extracts, respectively. The same authors also evaluated the antioxidant activity through the ferric reducing antioxidant power (FRAP) and oxygen radical absorbance capacity (ORAC) assays. The highest bioactivity was assigned to the leaf extracts and then to the flower extracts. These differences in the phenolic content may be justified by variations in edafoclimatic conditions of the locations where the samples were collected, which may affect the plant composition during the growing season [38]. Additionally, as verified in our study, the extraction method also causes significant variations in the evaluated responses. The used of different variables in obtaining hydromethanolic and decocted extracts, namely different solvents, extraction times, temperatures, and sample to solvent ratios, may have favored the extraction of different molecules.

Although nothing has been reported on the impact of gamma irradiation on the antioxidant activity or phenolic composition of *M. neglecta*, different effects have been reported in other plant materials. A study conducted by Pinela *et al.* [9] concluded that irradiation doses up to 10 kGy did not significantly affect the antioxidant activity and phenolic composition of decoctions and infusions prepared from *T. lignosa* samples. On the other hand, Pereira *et al.* reported increased antioxidant properties in infusions and methanolic extracts of borututu (*Cochlospermum angolensis* Welw.) [39] and *G. biloba* samples [25] irradiated at 10 kGy, respectively. The authors also verified that the 10 kGy dose improves the extractability of phenolic compounds from the *G. biloba* samples [40]. An increased total phenolic content and enhanced antioxidant activity of gamma irradiated almond skins extracted with 40% ethanol was reported by Harrison and Were [41]. Those authors attributed these results to the release of phenolic compounds from glycosidic components and degradation of larger phenolic compounds into smaller ones by the gamma irradiation treatment. In fact, the direct impact of gamma-rays and the indirect action of radiolytic products may change the structure of different antioxidant molecules and/or break some chemical bonds, thus leading to its decomposition or altered extractability from the plant material [15,25,41]. That is why the bioactivity of irradiated samples can either decrease or improve.

## 3. Materials and Methods

### 3.1. Dosimeters, Standards and Reagents

Amber Perspex routine dosimeters, Batch V, were purchased from Harwell Company (Oxfordshire, UK). Organic acids (oxalic, quinic, malic, citric, succinic and fumaric acids) and Trolox (6-hydroxy-2,5,7,8-tetramethylchroman-2-carboxylic acid) were purchased from Sigma (St. Louis, MO, USA). 2,2-Diphenyl-1-picrylhydrazyl (DPPH^•^) was obtained from Alfa Aesar (Ward Hill, MA, USA). All other chemicals and solvents were of analytical grade and purchased from common sources. Water was treated in a Model A10 Milli-Q water purification system (Millipore, Billerica, MA, USA).

### 3.2. Sample Collectionand Irradiation Experiments

Plant material (leafy flowering stems) from dwarf mallow (*Malva neglecta* Wallr. Fam. Malvaceae) was sustainably harvested from the wild in June at Miranda do Douro, Northeastern Portugal, considering local medicinal uses as well as healers’ and selected consumers’ criteria, which are related to particular gathering sites and requirements for safe herbal dosages forms, namely decoctions [42]. Taxonomic identification of the plant material was confirmed by Dr. Ana Maria Carvalho from the Polytechnic Institute of Bragança, Portugal. A voucher specimen was deposited in the Herbarium of the School of Agriculture of Bragança. Samples were then lyophilized (FreeZone 4.5, Labconco, Kansas City, MO, USA), reduced to a fine dried powder (20 mesh) and mixed to obtain a homogeneous sample.

The obtained powdered sample was divided into four portions and submitted to 1, 5 and 8 kGy of gamma-rays (predicted doses). A non-irradiated control (0 kGy) followed all the experiments. The irradiation was performed in a cobalt-60 experimental chamber (Precisa 22, Graviner Manufacturing Company Ltd., London, UK) located at the University of Lisbon Centre for Nuclear Sciences and Technologies (C2TN, Lisbon, Portugal), with four sources and a total activity of 177 TBq (4.78 kCi; January 2014). During the irradiation process, Amber Perspex routine dosimeters were used to measure the distribution of the absorbed energy and to determine the maximum (*D*_max_) and the minimum (*D*_min_) dose absorbed by the samples, following the procedure previously described by Fernandes *et al.* [43]. The measured average doses were 1.10 ± 0.16 kGy, 4.82 ± 0.10 kGy and 8.07 ± 0.46 kGy for the samples irradiated at the predicted doses of 1, 5 and 8 kGy, respectively. The estimated dose rate for the irradiation position, obtained with a Fricke dosimeter [43], was 1.9 kGy/h and the dose uniformity ratio (*D*_max_/*D*_min_) was 1.2. For simplicity, the predicted doses were considered in the text.

### 3.3. Color Measurement

The powdered samples were placed on a adapter for granular materials (model CR-A50, Konica Minolta Sensing, Inc., Sakai, Osaka, Japan) to reduce external interferences and data were collected in three different points on each set of samples with a colorimeter (model CR-400, Konica) previously calibrated using the standard white plate. Using illuminant C and the diaphragm opening of 8 mm, the CIE L*a*b* color space values were registered through the computerized system using the SpectraMagic Nx (version CM-S100W) color data software. Average values were considered to determine the color coordinates, where L* represents lightness, a* represents chromaticity on a green (–) to red (+) axis, and b* represents chromaticity on a blue (–) to yellow (+) axis. The total color difference (ΔE*) was calculated according to the CIEDE2000 equation [44].

### 3.4. Preparation of Decoctions and Hydromethanolic Extracts

Decoctions were prepared according to folk recipes/formulations [42]. Briefly, each powdered sample (1 g) was added to distilled water (200 mL) and boiled for 5 min. The mixture was left to stand at room temperature for 5 min more and then was filtered through Whatman No. 4 paper. The obtained decoctions were frozen and lyophilized.

Hydromethanolic extractions were performed by stirring each powdered sample (1 g) with methanol/water (30 mL, 80:20, *v*/*v*) at 25 °C and 150 rpm for 1 h. After filtering the supernatant through Whatman No. 4 paper, the residue was extracted with an additional portion (30 mL) of the hydromethanolic mixture. The combined extracts were concentrated under reduced pressure (R-210 rotary evaporator, Büchi, Flawil, Switzerland) and then frozen and lyophilized.

The lyophilized decocted and hydromethanolic extracts were redissolved in water and methanol/water (80:20, *v*/*v*), respectively, to obtain 4 mg/mL stock solutions which were successively diluted to different concentrations for evaluation of the antioxidant activity and total phenolics and flavonoids.

### 3.5. Analysis of Organic Acids

Organic acids were analyzed by ultra fast liquid chromatography (UFLC) coupled with photodiode array (PDA) detection according to the procedures previously described by Pereira *et al.* [28]. Briefly, the powdered samples (~1 g) were extracted by stirring with metaphosphoric acid (25 mL, 25 °C at 150 rpm) for 45 min and then subsequently filtering through Whatman No. 4 paper. For decoctions, the lyophilized extracts (~10 mg) were dissolved in metaphosphoric acid (1 mL). All samples were filtered through 0.2 μm nylon filters before analysis. The organic acids found were quantified by comparison of the area of their peaks recorded at 215 nm with calibration curves obtained from commercial standards of each compound. The results were expressed in mg per g of dry weight or lyophilized decoction (dw).

### 3.6. Evaluation of the Total Phenolic and Flavonoid Content

The total phenolic content was determined in the hydromethanolic and decocted extracts concentrated at 1.25 mg/mL by the Folin-Ciocalteu method [45,46] with slight modifications [47]. This assay is based on the formation of a blue-colored complex between the molybdenum and tungsten present in the Folin-Ciocalteu reagent upon reaction with reducing agents, which is monitored at 765 nm. The standard curve was calculated using gallic acid and the results were expressed as mg of gallic acid equivalents (GAE) per g of extract.

The total flavonoid content was determined in the hydromethanolic and decocted extracts concentrated at 2.5 mg/mL using the aluminium chloride colorimetric method [48] with slight modifications as described by the authors [49]. This assay is based on the formation of a flavonoid-aluminium complex, which is monitored at 510 nm. The standard curve was calculated using catechin and the results were expressed as mg of catechin equivalents (CE) per g of extract.

### 3.7. Evaluation of the Antioxidant Activity

The hydromethanolic and decocted extracts at different concentrations were submitted to four distinct *in vitro* assays to evaluate its antioxidant capacity [9]. Briefly, the DPPH^•^ scavenging activity and the reducing power assays were performed using an ELX800 Microplate Reader (Bio-Tek Instruments, Inc.; Winooski, VT, USA). The reduction of DPPH^•^ was determined after incubation of the different extracts with a methanolic solution containing DPPH^•^ (6 × 10^−5^ M) for 60 min in the dark by measuring the absorbance at 515 nm. The radical scavenging activity (RSA) was calculated as a percentage of DPPH^•^ discoloration using the Equation (1):

RSA (%) = [(A_DPPH_ − A_S_)/A_DPPH_] × 100 (1)

where A_DPPH_ is the absorbance of the DPPH^•^ solution and A_S_ is the absorbance of the solution containing the sample extract. The reducing power was evaluated by the ferricyanide/Prussian blue assay, whereby the capacity of the extracts to convert potassium ferricyanide (K_3_[Fe(CN)_6_]) into potassium ferrocyanide (K_4_[Fe(CN)_6_]), which then reacts with ferric chloride (FeCl_3_) to form a ferric-ferrous complex that is measuring spectrophotometrically, was monitored at 690 nm. The β-carotene bleaching inhibition (CBI) was evaluated by measuring the capacity of the extracts to neutralize linoleate free radicals. The different extracts were mixed with an emulsion containing β-carotene, linoleic acid and Tween 80 emulsifier and the absorbance was immediately measured at 470 nm in a Model 200 spectrophotometer (Analytik Jena, Jena, Germany). After 2 h of incubation at 50 °C, the absorbance was read again. The CBI was calculated using the Equation (2):

CBI (%)= (A_120_/A_0_) × 100 (2)

where A_120_ is the absorbance of the emulsion after 2 h of incubation and A_0_ is the initial absorbance. The thiobarbituric acid reactive substances (TBARS) formation inhibition was evaluated in porcine brain homogenates. The color intensity of the malondialdehyde-thiobarbituric acid (MDA-TBA) complex formed during heating of the reaction mixture at 80 °C was measured at 532 nm. The inhibition ratio was calculated using the Equation (3):

Inhibition ratio (%) = [(A − B)/A] × 100
(3)

where A and B correspond to the absorbance of the control and the sample solution, respectively. The results were expressed in EC_50_ values (mg/mL), *i.e.*, sample concentration providing 50% of antioxidant activity or 0.5 of absorbance in the reducing power assay. Trolox was used as positive control.

### 3.8. Statistical Analysis

In all cases, analyses were carried out using three samples separately processed, and all the assays were carried out in triplicate. The results were expressed as mean ± standard deviation and analyzed using one-way analysis of variance (ANOVA) followed by Tukey’s HSD test with *α* = 0.05. This treatment was carried out using IBM SPSS Statistics for Windows, Version 22.0 (IBM Corp., Armonk, NY, USA).

## 4. Conclusions

This study demonstrated the adequacy of gamma irradiation at 5 and 8 kGy to preserve the color parameters of *M. neglecta* dry material. Moreover, it was confirmed that the observed irradiation-induced modifications of color parameters not only depend on the applied dose, but also on the plant material under study. Oxalic, quinic, malic, citric, succinic and fumaric acids were identified in this plant for the first time. The total levels recorded in decocted extracts were always higher than those found in the plant dry material, as well as the individual levels of each organic acid (except for citric acid). Irradiation at 5 kGy increased the amounts of citric and succinic acids and decreased the fumaric acid levels in both matrices. In general, decoctions were preferred for their higher levels of total phenolics and flavonoids, DPPH^•^ scavenging activity and reducing power. In these preparations, the antioxidant properties and levels of total phenolics and flavonoids were decreased with the 8 kGy dose. In turn, the hydromethanolic extracts obtained from samples irradiated at 1 kGy showed decreased levels of total phenolic, total flavonoids, and lower antioxidant properties. Thus, decoctions were highlighted by interesting antioxidant properties and levels of total phenolics and organic acids. Nevertheless, further studies are of interest to evaluate the decontamination effectiveness of this technology and to investigate the effect in other quality parameters.

## Figures and Tables

**Table 1:** Organic acids content in the dry material and decocted extracts of *M. neglecta* samples submitted to gamma irradiation.

Dose	Oxalic Acid	Quinic Acid	Malic Acid	Citric Acid	Succinic Acid	Fumaric Acid	Total
**Dry material (mg/g dw)**							
**0 kGy**	67.50 ± 0.16 ^b^	6.63 ± 0.21 ^b^	3.16 ± 0.28 ^b^	21.56 ± 0.41 ^b^	7.50 ± 0.13 ^a^	15.07 ± 0.03 ^c^	121.43 ± 1.17 ^b^
**1 kGy**	67.29 ± 0.05 ^b^	7.50 ± 0.42 ^a^	3.09 ± 0.16 ^b^	19.84 ± 0.14 ^c^	7.30 ± 0.41 ^a^	18.04 ± 0.05 ^a^	123.07 ± 0.02 ^a^
**5 kGy**	65.30 ± 0.17 ^c^	6.34 ± 0.28 ^b^	3.42 ± 0.33 ^ab^	22.28 ± 0.31 ^a^	7.29 ± 0.12 ^a^	13.59 ± 0.07 ^d^	118.22 ± 1.15 ^c^
**8 kGy**	71.75 ± 0.10 ^a^	6.45 ± 0.08 ^b^	3.64 ± 0.05 ^a^	19.02 ± 0.08 ^d^	6.23 ± 0.13 ^b^	15.85 ± 0.05 ^b^	122.94 ± 0.30 ^ab^
**Decocted extracts (mg/g dw)**							
**0 kGy**	106.71 ± 0.50 ^a^	3.59 ± 0.25 ^d^	9.78 ± 0.67 ^b^	7.92 ± 0.09 ^d^	47.43 ± 0.67 ^b^	20.61 ± 0.14 ^c^	196.03 ± 0.80 ^b^
**1 kGy**	79.80 ± 0.05 ^c^	6.96 ± 0.20 ^c^	10.52 ± 0.08 ^a^	9.80 ± 0.15 ^b^	47.36 ± 0.38 ^b^	21.54 ± 0.19 ^b^	175.99 ± 0.89 ^c^
**5 kGy**	88.72 ± 0.03 ^b^	8.90 ± 0.09 ^a^	9.72 ± 0.19 ^b^	12.76 ± 0.07 ^a^	54.17 ± 0.06 ^a^	20.81 ± 0.08 ^c^	195.09 ± 0.01 ^b^
**8 kGy**	106.33 ± 0.10 ^a^	8.37 ± 0.39 ^b^	7.89 ± 0.29 ^c^	8.58 ± 0.35 ^c^	41.42 ± 0.50 ^c^	25.17 ± 0.09 ^a^	197.76 ± 0.95 ^a^

The results are presented as mean ± standard deviation. In each column and for each sample (dry material and decoction), different letters (a–d) indicate statistically significant differences (*p* < 0.05).

**Table 2:** Antioxidant activity of the hydromethanolic and decocted extracts prepared from the *M. neglecta* dry material submitted to gamma irradiation.

Dose	DPPH^•^ Scavenging Activity (EC_50_ values, mg/mL)	Reducing Power (EC_50_ values, mg/mL)	β-Carotene Bleaching Inhibition (EC_50_ values, mg/mL)	TBARS Formation Inhibition (EC_50_ values, mg/mL)	Total Phenolics (mg GAE/g Extract)	Total Flavonoids (mg CE/g Extract)
***Hydromethanolic extracts***						
**0 kGy**	1.15 ± 0.02 ^c^	0.52 ± 0.01 ^c^	0.46 ± 0.01 ^a^	0.56 ± 0.05 ^b^	69.54 ± 0.21 ^b^	22.85 ± 0.52 ^b^
**1 kGy**	1.57 ± 0.02 ^b^	0.69 ± 0.01 ^a^	0.41 ± 0.03 ^b^	0.58 ± 0.03 ^a^	55.04 ± 0.36 ^d^	19.56 ± 0.08 ^c^
**5 kGy**	1.06 ± 0.07 ^d^	0.57 ± 0.02 ^b^	0.41 ± 0.01 ^b^	0.11 ± 0.03 ^d^	78.55 ± 0.67 ^a^	27.30 ± 0.20 ^a^
**8 kGy**	1.75 ± 0.04 ^a^	0.56 ± 0.01 ^b^	0.40 ± 0.01 ^b^	0.22 ± 0.01 ^c^	64.78 ± 1.51 ^c^	22.70 ± 0.51 ^b^
***Decocted extracts***						
**0 kGy**	0.37 ± 0.01 ^c^	0.268 ± 0.002 ^b^	0.16 ± 0.01 ^c^	0.403 ± 0.004 ^b^	91.05 ± 1.14 ^b^	25.14 ± 0.53 ^b^
**1 kGy**	0.40 ± 0.01 ^b^	0.264 ± 0.003 ^c^	0.17 ± 0.02 ^c^	0.35 ± 0.01 ^c^	96.92 ± 3.73 ^a^	28.03 ± 0.07 ^a^
**5 kGy**	0.36 ± 0.01 ^c^	0.253 ± 0.003 ^d^	0.41 ± 0.01 ^b^	0.32 ± 0.01 ^d^	96.76 ± 0.60 ^a^	27.63 ± 0.35 ^a^
**8 kGy**	0.46 ± 0.02 ^a^	0.326 ± 0.001 ^a^	0.46 ± 0.01 ^a^	0.69 ± 0.02 ^a^	78.99 ± 0.30 ^c^	21.98 ± 0.47 ^c^

The results are presented as mean ± standard deviation. In each column and for each sample (hydromethanolic and decocted extracts), different letters (a–d) indicate statistically significant differences (*p* < 0.05). EC_50_: Extract concentration corresponding to 50% of antioxidant activity or 0.5 of absorbance in the reducing power assay. Trolox EC_50_ values: 42 µg/mL (DPPH**^•^** scavenging activity), 41 µg/mL (reducing power), 18 µg/mL (β-carotene bleaching inhibition) and 23 µg/mL (TBARS inhibition). GAE: gallic acid equivalents; CE: catechin equivalents.
